# Absence of *Batf3* results in reduced liver pathology in mice infected with *Schistosoma japonicum*

**DOI:** 10.1186/s13071-017-2250-1

**Published:** 2017-06-24

**Authors:** Lin Chen, Donghui Zhang, Wenyue Zhang, Yuxiao Zhu, Min Hou, Bingya Yang, Zhipeng Xu, Minjun Ji, Guanling Wu

**Affiliations:** 1 Department of Pathogen Biology, Nanjing Medical University, Nanjing, Jiangsu China; 2 Jiangsu Province Key Laboratory of Modern Pathogen Biology, Nanjing, Jiangsu China; 3 School of International Education, Nanjing Medical University, Nanjing, Jiangsu China; 4 Institute of Dermatology, Chinese Academy of Medical Sciences and Peking Union Medical College, Nanjing, China

**Keywords:** *Schistosoma japonicum*, Dendritic cells, CD8α^+^, CD8^+^T cells, *Batf3*, Helminth

## Abstract

**Background:**

The involvement of CD8^+^T cells in schistosomiasis is being increasingly appreciated, but the underlying mechanism is not well defined.

**Results:**

In this study, we showed that the absence of *Batf3* alleviated liver damage in *Batf3* ^−/−^ mice infected with *S. japonicum.* We found alleviated liver granulomatous inflammation in *Batf3* ^−/−^ mice with schistosomiasis japonica could not be attributed to the difference in schistosome egg or worm burden. The stronger Tc1 cell responses observed in *Batf3* ^−/−^ mice suggested that the deletion of *Batf3* resulted in more activation of CD8^+^T cells unexpectedly during the natural infection of schistosomes. We detected a small amount of CD8α^+^ DCs in the spleen of *Batf3* ^−/−^ mice at 9w post-infection. This small amount of newly generated CD8α^+^ DCs might contribute to enhanced activation of CD8^+^T cells via cross-presentation and activation which then attenuate hepatic pathological damage found in *Batf3* ^−/−^ mice.

**Conclusions:**

Our study provides evidence that *Batf3* is associated with the immunoregulation of the liver granuloma formation, which may confer a new options for schistosomiasis treatment.

## Background

A challenging problem in the prevention and treatment of schistosomiasis is re-infection and immunopathological damage of the liver in patients who live in endemic areas [1, 2].The secretion of soluble egg antigen (SEA) in the late stage of schistosomiasis causes Th2-dominant immune response to form granulomas [3, 4]. Repeated infection will eventually lead to secondary liver fibrosis and advanced schistosomiasis, and patients may have upper gastrointestinal bleeding, hepatic coma and other serious complications, and even death [2].

Current studies on the immune mechanism of schistosomiasis infection have focused on CD4^+^T cells and antibody response. CD8^+^T cells play an important role in host defense against bacterial, viral, protozoa infection and anti-tumor processes, however, the role of CD8^+^T cells in parasitic helminth infection, such as schistosomiasis, remains unclear. CD8^+^T cells recognize the antigenic peptide presented by the MHCI molecule and kill the target cells, or induce apoptosis of the target cells by direct contact (secretion of perforin, granzyme, lymphotoxin, or by Fas / FasL pathway). Schistosomes can adsorb host MHCI, suggesting that host CTL may play a role in the immune response to multicellular worms [5]. Chensue et al. [6] proposed that modulated granulomatous inflammatory response was maintained by effector and regulator lymphocytes in mice with chronic infection of *Schistosoma mansoni*. Among those studied, Ly-2^+^ (CD8^+^) T lymphocytes were primarily responsible for suppressing granuloma formation. Another study [7] found that during the chronic infection stage of *S. mansoni*, CD8^+^ T cell activity increased, which can significantly reduce the growth of egg granuloma, and the subsequent formation of new granulomas was significantly slower at 16–20 weeks post-infection. When lymph nodes and spleen cells in chronic stage of infection were transferred to acutely infected mice, they can significantly reduce granuloma formation in the recipients. However, when the CD8^+^ T cells were removed from these cells, the inhibition effect on granuloma disappeared [8]. Pancré et al. [9, 10] reported that single immunization of *S. mansoni* recombinant glutathione S-transferase (rSm28GST) induced the immunity to the infection of schistosomiasis and increased spleen CD8^+^T cell activity, resulting in antigen-specific CTL response. Sm28GST-specific CD8^+^T cells were further passively transferred to naive mice, after the infection of schistosomiasis, the number of liver granuloma reduced, and liver fibrosis alleviated in the mice. However, after treatment with anti-CD8 antibody, the protective effect mediated by Sm28GST was significantly reducedx. Consistent with the above studies, our previous study in *TLR2* ^*−/−*^, *TLR4* ^*−/−*^ mice and pigs suggest that CTL responses are involved in the immune mechanism of resistance to *S. japonicum* infection [11, 12].

Moreover, CD8^+^T cells secrete inflammatory factor IFN-γ to regulate immune response and reduce pathological damage during the infection of *S. mansoni*. CD8^+^T cells in the spleen of mice infected with *S. mansoni* responded to schistosome antigen presented by APC and produced IFN-γ [13]. After *S. mansoni* infection, 4 weeks of IFN-γ intramuscular injection reduced collagen deposition in mouse liver significantly [14]. In the spleen of mice infected with *S. mansoni*, the number of CD8^+^T cells decreased, type 2 immune environment established and further led to the apoptosis of Tc1(CD3^+^CD8^+^IFN-γ^+^) cell. Thus, schistosomes may inhibit type 1 immune response by reducing Tc1 cells to facilitate the deposition of eggs and cause pathological damage [15].

Taken together, CD8^+^T cells may play an immunological role in anti-schistosomiasis infection. However, the mechanism by which CD8^+^T cell response is generated in schistosome infection remains unclear. In this study, we found the absence of *Batf3* alleviated liver damage in *Batf3* ^−/−^ mice infected with *S. japonicum* and that the alleviated liver granulomatous inflammation could not be attributed to the difference in schistosome egg or worm burden. The stronger Tc1 cell responses observed in *Batf3* ^−/−^ mice suggested that the deletion of *Batf3* activated CD8^+^T cells unexpectedly during the natural infection of schistosomes. Then we detected a small amount of CD8α^+^ DCs in the spleen of *Batf3* ^−/−^ mice at 9w post-infection. This small amount of newly generated CD8α^+^ DCs may have a more powerful function in cross-presenting and activate CD8^+^T to secret IFN-γ^+^ which can attenuate hepatic pathological damage in *Batf3* ^−/−^ mice.

## Methods

### Mice

B6.129S(C)-*Batf3* ^*tm1Kmm*^/J mice (Stock No: 013755/*Batf3* ^−/−^) were purchased from Jackson Labs [16]. Wild-type C57BL/6 J (B6) mice were purchased from the Model Animal Research Center, Nanjing University (Nanjing, China). 6–8 weeks old female mice were used in all experiments. All mice were maintained according the institutional guidelines at Nanjing Medical University.

### Parasites and infections

*Schistosoma japonicum* cercariae were maintained in *Oncomelania hupensis* snails (laboratory-infected with a Chinese mainland strain), which were purchased from the Jiangsu Institute of Parasitic Disease (Wuxi, China). Snails were placed in deionized water and exposed to incandescent light for 3–4 h for cercarial release. For infection, the cercariae were counted and placed on glass cover slips by a 10 μl bacteriological loop. *Batf3* ^−/−^ and B6 mice were infected with 10 ± 2 *S. japonicum* cercariae through their shaved abdomens.

### Parasitological assessments

For parasite burden determination (adult worm recovery, egg burdens and area of single egg granuloma in the livers), all mice were sacrificed 9 weeks post-infection. The worms were collected and counted through perfusion of the portal vein with PBS. After perfusion, the intestinal tract of each mouse was examined for residual worms. The liver samples, except left front lobes, of each mouse were weighted and digested in 5% KOH for 18 h at 37 °C. Each liver sample was counted 3 times for released eggs under the microscope and the mean count was used as eggs per gram (EPG) in mice. Left front lobes of livers from each mouse were fixed in 4% paraformaldehyde, embedded in paraffin and stained with haematoxilin and eosin according to standard protocols. Single-egg granulomas were examined and their sizes were calculated using AxioVision Rel 4.7 (Carl Zeiss GmbH, Jena, Germany). At least 10 single egg granulomas per liver section were photographed. Sirius red stainings were observed to investigate the deposition of collagen fibers in liver of *Batf3* ^−/−^ and B6 mice at 9 weeks post-infection and the results were analyzed using imageJ software (National Institutes of Health, America).

### Flow cytometry detection

Percentages of Th1 (CD3^+^CD4^+^IFN-γ^+^), Th2 (CD3^+^CD4^+^IL-4^+^), Tc1 (CD3^+^CD8^+^IFN-γ^+^), Tc2 (CD3^+^CD8^+^IL-4^+^) cells in the spleens of *Batf3* ^−/−^ and B6 mice at 0, 3, 6 and 9 weeks post-infection was detected by flow cytometry. Splenocytes were prepared by gently forcing spleen tissue through a nylon net into incomplete RPMI-1640 medium (Gibco-Invitrogen, Grand Island, NY, USA) supplemented with 100 U/ml penicillin and 100 U/ml streptomycin (Gibco-Invitrogen), followed by red blood cell (RBC) lysis to remove erythrocytes. Then 2.0 × 10^6^ splenocytes cells were stimulated with ionomycin (1 μg/ml) and PMA (25 ng/ml) in the presence of 10 μg/ml Brefeldin-A (Enzo Life Science, New York, USA) for 6 h at 37 °C in 5% CO_2_. After 6 h, the cells were surface stained with APC-anti-CD3e, FITC-anti-CD4 (or CD8). Subsequently, the cells were washed, fixed and permeabilized with Cytofix/Cytoperm buffer and stained with PE conjugated antibodies against IFN-γ or IL-4 (or isotype IgG2a control antibody) (eBioscience, San Diego, CA, USA) following the manufacturer’s instruction. Stained cells were detected by flow cytometry (Becton Dickinson) and the data were analyzed using FlowJo7.6 software.

### Real-time polymerase chain reaction

Total RNA was extracted from 1 × 10^6^ splenocytes using TRIzol reagent (Invitrogen, Life Technologies Carlsbad, CA, USA). The cDNA was synthesized with PrimeScript RT reagent kit (Takara, Otsu, Shiga, Japan) according to the manufacturer’s protocol. PCR was performed on the ABI PRISM 7300 (Applied Biosystems, USA) using Power SYBR Green PCR Master Mix (Applied Biosystems, USA). Primers specific for *β-actin*, *Batf3*, *Irf8* and *PU.1* are listed in Table 1. PCR cycling protocol was as follows: 50 °C for 2 min, 95 °C for 10 min, followed by 40 cycles at 95 °C for 15 s, 60 °C for 1 min. The housekeeping gene *β-actin* was used as an internal control and the data were analyzed with 7300 System SDS Software v1.2.1 (Applied Biosystems, USA). Quantitation of relative mRNA expression was calculated using the 2^−ΔΔCt^ method [17].

**Table 1:** Primer sequences of *Irf8*, *PU.1*, *Batf3*, *Id2, Nfil3* genes used in the RT-PCR

		sequence (5'→3')
*β-actin*	sense	TTCCTTCTTGGGTATGGAAT
	antisense	GAGCAATGATCTTGATCTTC
*Irf8*	sense	GGGTCAGTACACAACAGGGG
	antisense	CTAGCTGCGTGGAGCATGTA
*PU.1*	sense	CCTCGATACTCCCATGGTGC
	antisense	GGCTGGGGACAAGGTTTGAT
*Batf3*	sense	TTTGTGCAGCTTCGGTCAGA
	antisense	CCGGACAAAGGAGGAGTGAG
*Id2*	sense	CGGGGCTGATCTGGGAAAAT
	antisense	CACAGCGTAACCTCGTCTTC
*Nfil3*	sense	ATGTTACAGGCGTGCAAAATGG
	antisense	TGATCGCTATGGCTTTCTCCA

### Statistical analysis

Statistical analyses were performed using GraphPad Prism version 5.01 for Windows(USA, GraphPad Software). The data are expressed as mean ± SEM. Student’s *t*-test and one-way analysis of variance (ANOVA) were performed to test for differences. For all tests, significance was considered for *P* < 0.05.

## Results

### The number of CD8α^+^ DCs changed consistently with the trend of Th1 response during the infection with *S. japonicum*

**Fig. 1:**
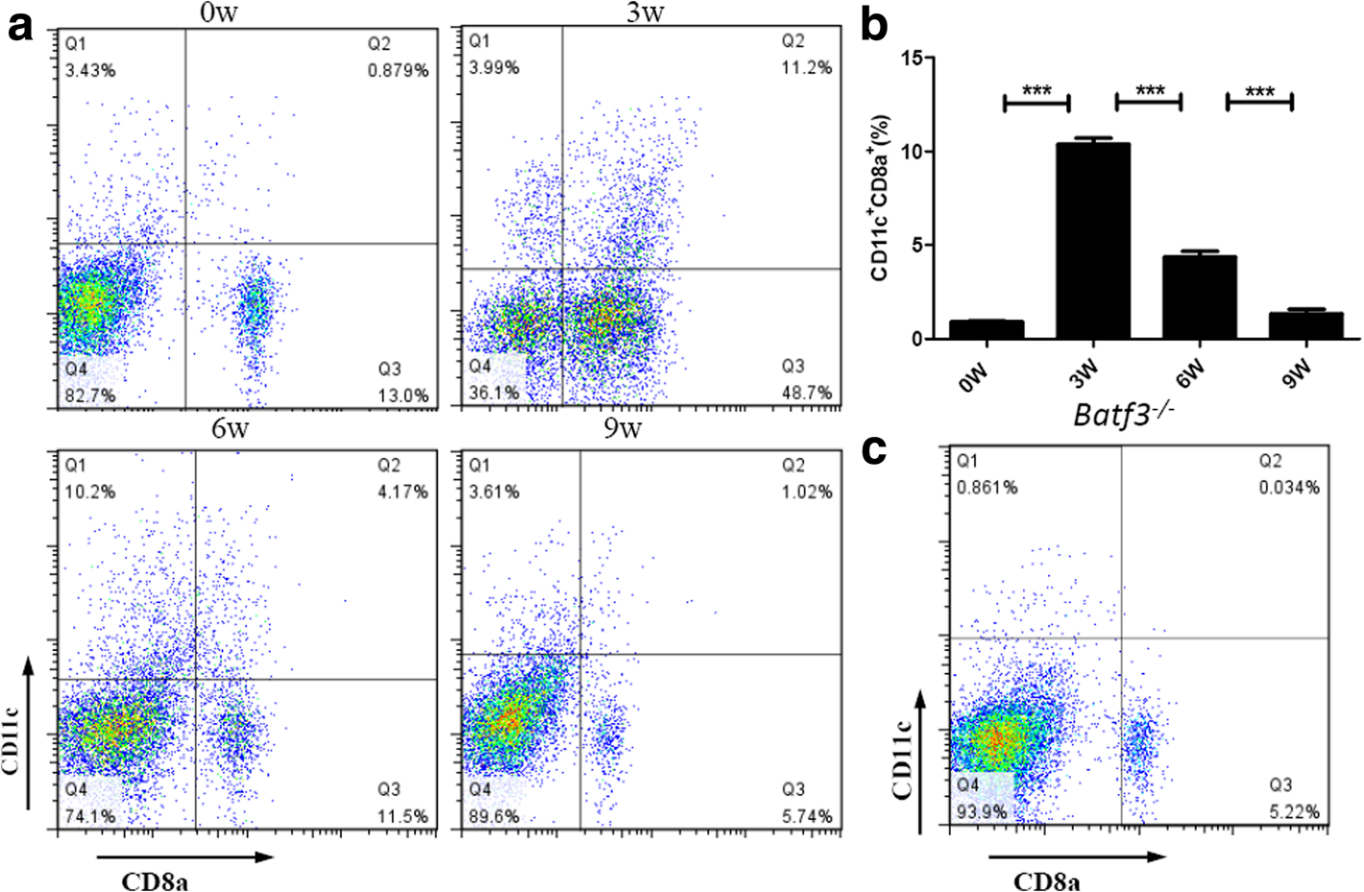
Dynamic changes of splenic dendritic cells subsets in mice infected with *S. japonicum*. The number of CD8α^+^DCs changed consistently with the trend of Th1 response during the infection of *S. japonicum*. **a** Percentages of CD11c^+^CD8a^+^ cells analysed by FACS at 0, 3, 6 and 9 weeks post-infection. The upper right quadrant is the proportion of CD8α^+^DCs subsets. **b** Percentage of CD8α^+^DCs in the spleen. Data are presented as the means ± SEM from six mice in each group (***P < 0.001). Results are representative of two independent experiments. **c** Percentages of CD11c^+^CD8a^+^ cells analysed by FACS in *Batf3* ^−/−^ mice. The upper right quadrant is the proportion of CD8α^+^DCs subsets

We established mouse model of schistosomiasis infection in B6 mice and detected the number of CD8α^+^DCs in the spleen at 0, 3, 6 and 9 weeks post-infection. We found that the number of CD8α^+^DCs increased quickly after infection and reached a peak at 3 weeks after infection, beginning to decrease from 6 weeks post-infection to the lowest point at 9 weeks post-infection (*F*_(3,12)_ = 300.9, *P* < 0.0001) (Fig. 1). This change was consistent with the trend of Th1 response during *S. japonicum* infection. In the initial 2–4 weeks after infection, under the stimulation of migrating schistosomula, the host showed Th1 type (IFN-γ, TNF) response. Once egg production begins, Th1 type response declined rapidly, and Th2 type (IL-4, IL-13, IL-10, IL-5) response started [18, 19].

### *Batf3* null mutation alleviates mouse liver granulomatous inflammation in *S. japonicum* infection

**Fig. 2:**
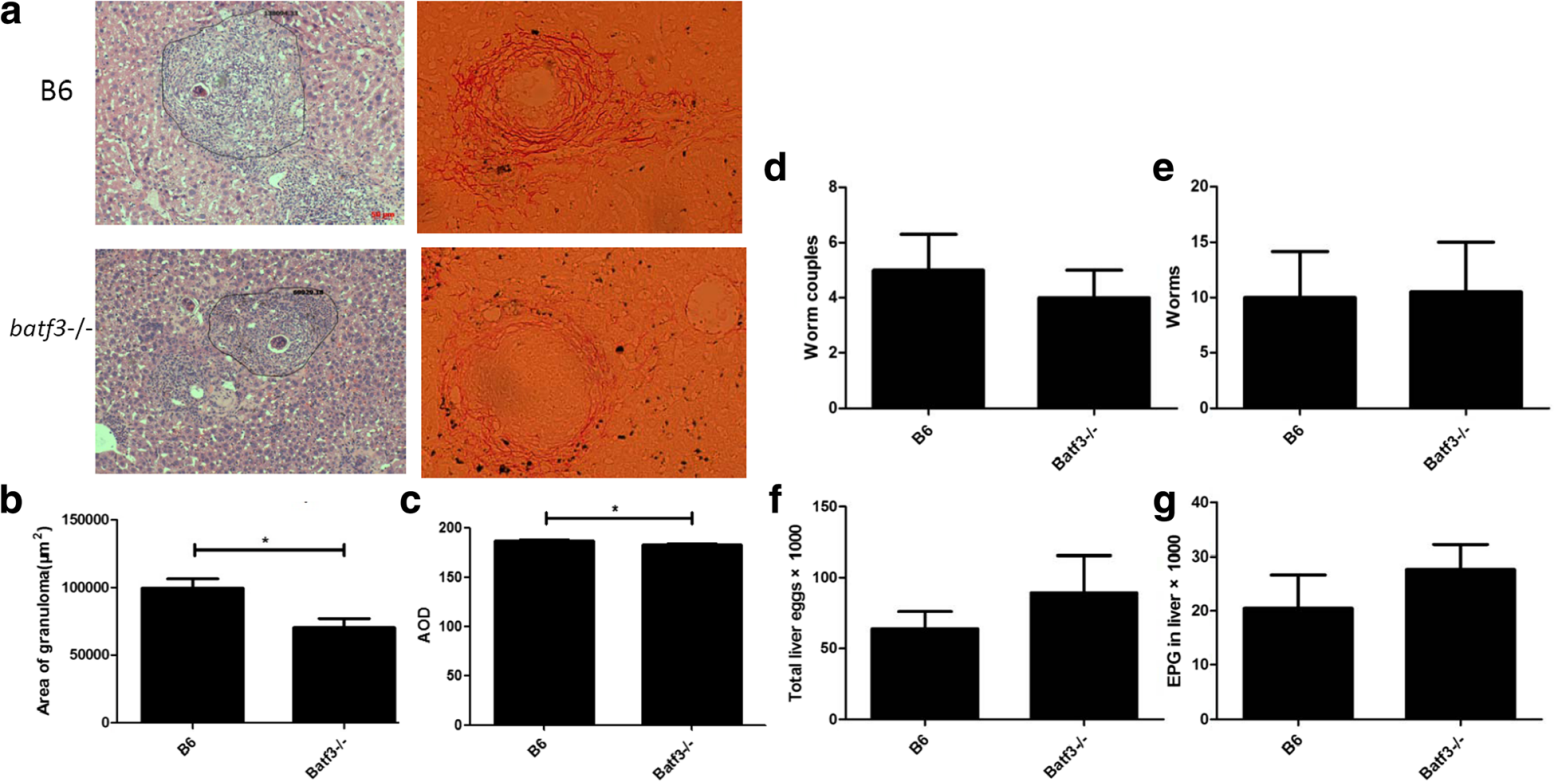
Parasite burden were observed at 9 weeks post *S. japonicum* infection in *Batf3* ^−/−^ and B6 mice. Infection by *S. japonicum* results in an alleviated liver granulomatous inflammation in *Batf3* ^−/−^mice. Worm and egg burdens are similar in *Batf3* ^−/−^ and B6 mice infected with *S. japonicum*. **a** Representative granulomas with a single egg from *Batf3* ^−/−^ and B6 group. Liver sections were stained with HE and sirius red for microscopic examination. **b** Average area of single egg granulomas from *Batf3* ^−/−^ and B6 group. Sizes of the granulomas were measured by computer-assisted morphometric analysis. **c** The average integrated optical density (AOD) in *Batf3* ^−/−^ and B6 mice. Results of deposition of collagen fibers were analyzed using imageJ software. **d** Average number of worm couples recovered. **e** Average number of worms recovered. **f** Total number of eggs in the liver. **g** Average number of eggs per gram (EPG) in the liver. Results are representative of two independent experiments. Each bar represents the mean ± SEM from six mice per group. (*P < 0.05). *Scale-bars*: 50 μm

To evaluate the role of CD8α^+^ DCs in schistosome infection, we used *Batf3* ^−/−^ mice which lack splenic CD8α^+^DCs, because of the deletion of *Batf3* (Fig. 1c), to establish a schistosome infection model. We found that the granulomas developed after the deposition of parasite eggs in the livers of both *Batf3* ^−/−^ and WT control B6 mice. The average size of liver granuloma in *Batf3* ^−/−^ mice was significantly smaller than that in the B6 mice at 9 weeks post-infection (*t*_(24)_ = 2.952, *P* = 0.0121, Fig. 2a, b). The average integrated optical density (AOD) in *Batf3* ^−/−^ mice was significantly lower than that in the B6 mice at 9 weeks post-infection (*t*_(21)_ = 2.141, *P* = 0.0441, Figs. 2a, c) which indicated less deposition of collagen fibers in liver of *Batf3* ^−/−^mice. These data suggest that CD8α^+^ DCs may be involved in the regulation of the granulomatous response to *S. japonicum* infection.

### Similar worm and egg burdens in *Batf3*^−/−^ and B6 mice infected with *S. japonicum*

After infection, schistosomula developed into adults and laid eggs; matured schistosome miracidium within eggs can secrete soluble egg antigen (SEA) and cause granulomatous response [20]. To clarify whether the difference of liver granulomatous inflammation between *Batf3* ^−/−^ and B6 mice was caused by the different worm and egg burdens, we evaluated parasite burdens of both groups at 9 weeks post-infection and the results showed that there were no significant differences in the numbers of paired worms (*t*_(12)_ = 0.4924, *P* = 0.6483) (Fig. 2d), adult worms (*t*_(12)_ = 0.07892, *P* = 0.9421) (Fig. 2e), total liver eggs (*t*_(12)_ = 0.8675, *P* = 0.4109) (Fig. 2f) and EPG in the liver (*t*_(12)_ = 0.9388, *P* = 0.3753) (Fig. 2g) between *Batf3* ^−/−^ and B6 mice. These results indicate that the alleviated liver granulomatous inflammation in *Batf3* ^−/−^ mice with schistosomiasis japonica is caused by other mechanisms rather than the difference in schistosome egg or worm burden.

### Tc1 cell responses are stronger in *S. japonicum* infected *Batf3*^−/−^ mice

**Fig. 3:**
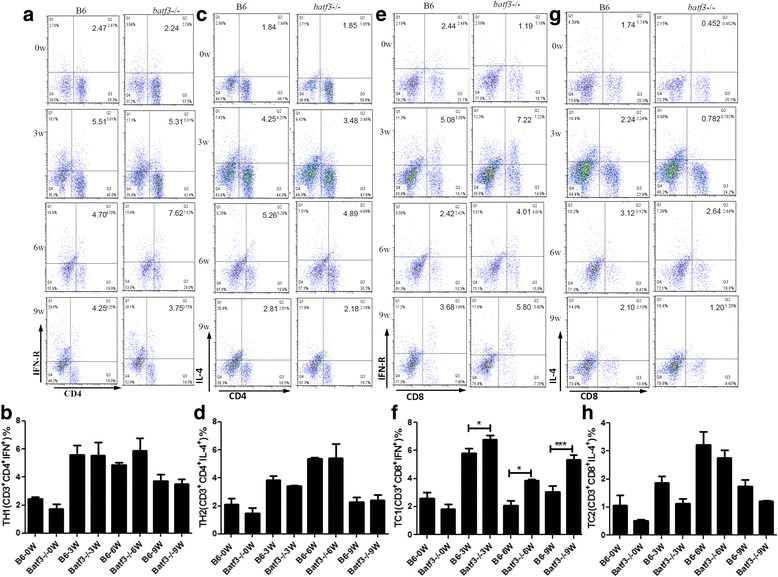
Immune responses of *Batf3* ^−/−^ and B6 mice after *S. japonicum* infection. Th1, Th2, Tc1, and Tc2 immune response were detected by FCM at 0, 3, 6 and 9 weeks post-infection of each group. Tc1 cell responses are stronger in *S. japonicum* infected *Batf3* ^−/−^ mice, Th1, Th2, and Tc2 cell responses show no significant difference between *Batf3* ^−/−^ and WT B6 mice after *S. japonicum* infection. **a**, **b** Percentages of CD3^+^CD4^+^IFN-γ^+^ (Th1) gated from CD3 + T cells analysed by FCM. **c**, **d** Percentages of CD3^+^CD4^+^IL-4^+^ (Th2) gated from CD3 + T cells analysed by FCM. **e**, **f** Percentages of CD3^+^CD8^+^IFN-γ^+^ (Tc1) gated from CD3 + T cells analysed by FCM. **g**, **h** Percentages of CD3^+^CD8^+^IL-4^+^ (Tc2) gated from CD3^+^T cells analysed by FCM. Results are representative of two independent experiments. Each bar represents the mean ± SEM from six mice per group. (*P < 0.05, **P < 0.01)

After infection, migrating schistosomula triggered Th1 polarization, which can downregulate hepatic granuloma formation by secreting INF-γ [21, 22]. Egg production can change the immune response to a bias of Th2 (caused by SEA), which can promote the liver lesion [21, 23]. Thus, we detected Th1 (CD3^+^CD4^+^IFN-γ^+^), Th2 (CD3^+^CD4^+^IL-4^+^), Tc1 (CD3^+^CD8^+^IFN-γ^+^) and Tc2 (CD3^+^CD8^+^IL-4^+^) cell responses in *Batf3* ^−/−^ and B6 mice by flow cytometry. During the first 3 weeks post-infection the percentage of Th1 cells in the spleen increased quickly in both *Batf3* ^−/−^ and B6 mice and then decreased from 6 weeks post-infection, but there was no significant difference in Th1 cells responses between these two groups (*F*_(7,15)_ = 0.01368, *P* = 0.9080) (Fig. 3a, b). During the first 3 weeks post-infection the percentage of Th2 cells in the spleen increased slowly in both *Batf3* ^−/−^ and B6 mice, but the proportion of Th2 cells in both *Batf3* ^−/−^ and B6 mice experienced a faster increase after 3 weeks, although there was still no significant difference between these two groups (*F*_(7,14)_ = 0.02747, *P* = 0.8700) (Fig. 3c, d). In addition, the percentage of Tc2 cells in the spleen of both *Batf3* ^−/−^ and B6 mice increased slowly since infection, and then rapidly increased to the top level at 6 weeks post-infection. However, there was no significant difference between these two groups (*F*_(7,15)_ = 1.540, *P* = 0.2283) (Fig. 3g, h). Figure 3e and f shows that in the first 3 weeks post-infection, the increase of the percentage of Tc1 cells in the spleen of both *Batf3* ^−/−^ and B6 mice was accelerated and then decreased from 3 weeks post-infection. Notably, Tc1 cells in *Batf3* ^−/−^ mice were more than those in B6 control mice at 3, 6 and 9 weeks post-infection (*F*_(7,15)_ = 30.85, *P* < 0.0001) (Fig. 3e, f). These results suggest a correlation between the lack of CD8α^+^ DCs and increased generation of Tc1 cells during *S. japonicum* infection.

### Tc1 cell responses induced by *Batf3* independent CD8α^+^ DCs

**Fig. 4:**
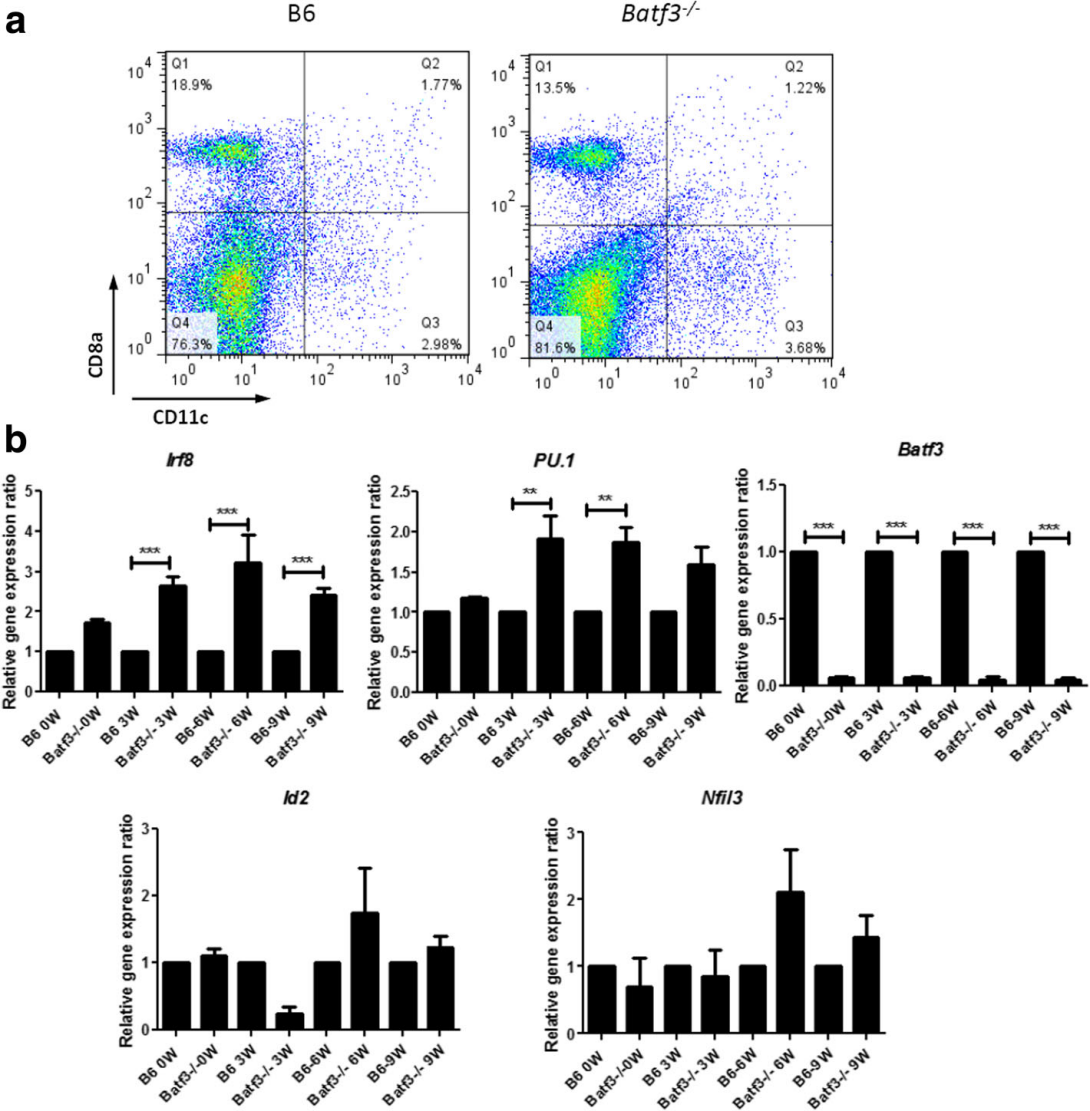
Tc1 cell responses induced by *Batf3*-independent CD8α^+^ DCs. **a** A small amount of CD8α^+^ DCs was detected in the *Batf3* ^−/−^ mouse schistosomiasis infection model.Percentages of CD11c^+^CD8a^+^cells analysed by FCM at 9 weeks post-infection. The upper right quadrant is the proportion of CD8α^+^ DCs cell subsets. **b** Expression of *Irf8*, *PU.1*, *Id2, Nfil3* and *Batf3*genes of *Batf3* ^−/−^ and B6 mice after *S. japonicum* infection. Expression of *Irf8*, *PU.1*, *Id2, Nfil3* and *Batf3* genes in the spleen were detected at 0, 3, 6 and 9 weeks post-infection by RT-PCR. (**P < 0.01, ***P < 0.001) Results are representative of two independent experiments. Data are presented as the mean ± SEM from six mice in each group

The stronger Tc1 cell responses observed in *Batf3* ^−/−^ mice suggested that the deletion of *Batf3* activated CD8^+^T cells unexpectedly during the natural infection of schistosome. It is of interest how CD8^+^T cells were activated, since there are no CD8α^+^ DCs in the spleen of uninfected *Batf3* ^−/−^ mice (Fig 1c). Surprisingly, there was still a small amount of CD8α^+^ DCs in the spleen of *Batf3* ^−/−^ mice at 9 weeks post-infection (Fig. 4a). It was reported that CD8α^+^DCs differentiation is regulated by a series of transcriptional regulators [16, 24]. We then detected the expression levels of *Irf8*, *PU.1*, *Id2, Nfil3* and *Batf3* in the spleen of *Batf3* ^−/−^ and B6 mice. Compared to wild-type B6 mice, *Irf8* expression in *Batf3* ^−/−^ mice was higher at 3w, 6w and 9w (*F*_(7,15)_ = 21.12, *P* < 0.0001). The expression of *PU.1* significantly increased at 3w and 6w (*F*_(7,15)_ = 7.860, *P* = 0.0004) (Fig. 4b). However, there were no significant differences in expression levels of *Id2* and *Nfil3* between *Batf3* ^−/−^ and B6 mice (*F*_(7,15)_ = 2.635, *P* = 0.0514; *F*_(7,15)_ = 1.970, *P* = 0.1281, respectively) (Fig. 4b). Our results suggest that in the absence of *Batf3*, a small amount of *Batf3-*independent CD8α^+^ DCs might be compensatorily induced in an *Irf8*-dependent manner.

## Discussion

APC recognizes the endogenous antigen and processes it as antigen peptide-MHCI complex to CD8^+^T cells to induce CD8^+^T cell response, while it recognizes the exogenous antigens and processes it as antigen peptide-MHCII complex to CD4^+^T cells to induce Th cell response [25]. The cross-presentation theory provides a basis for the induction of CD8^+^T responses during extracellular infection. Dendritic cells are known to be the most powerful APC, and connect innate and adaptive immune responses. The spleen immature DCs of mice can be divided into CD8α^+^DCs and CD8α^−^DCs, according to surface molecular markers [26]. Studies have demonstrated that CD8α^+^DCs play a unique role in cross-presenting [27–29].

In this study, we found that the number of CD8α^+^DCs in the spleen of mice infected with *S. japonicum* has the same trend of change with Tc1 response, suggesting that CD8^+^T cell response in schistosomiasis infection is likely to be related to the cross-presentation of CD8α^+^ DCs. CD8α^+^DCs differentiation is regulated by a series of transcriptional regulators, including interferon regulatory factor 8 (*Irf8*), nuclear factor interleukin 3 (*Nfil3*), DNA binding inhibitor of DNA binding 2 (*Id2*) [24, 30–32], and basic leucine zipper transcriptional factor ATF-like 3(*Batf3*) [16, 24]. Previous studies suggest that the *Batf3* plays a key role in the differentiation of CD8α^+^ DCs, *Batf3* ^−/−^mice may lose the ability to cross-present cell-associated antigens and soluble antigens due to the loss of CD8α^+^ DCs [16, 33, 34]. Hildner et al. [16] reported that *Batf3* ^−/−^ mice lack virus-specific CD8^+^T cells when infected with West Nile virus, and *Batf3* ^−/−^ mice were more likely to develop fibrosarcoma tumor models than wild-type mice because of a lack of CD8^+^T cell responses. Torti et al. [34] reported a severe deletion of MCMV-specific CD8^+^T cells in *Batf3* ^−/−^ mice. Mashayekhi et al. [35] found that *Batf3* ^−/−^ mice were more likely to be infected by *Toxoplasma gondii* due to the lack of CD8α^+^ DCs and decreased IFN-γ and IL-12 production.

To evaluate the role of CD8α^+^ DCs s in schistosome infection, we infected *Batf3* ^−/−^ and wild-type B6 mice by *S. japonicum* cercariae. Our results showed that *Batf3* ^−/−^ mice had significantly smaller average size of granuloma in the liver, not because of the difference in schistosome egg or worm burden, suggesting that CD8α^+^ DCs may be involved in the regulation of the granulomatous response during *S. japonicum* infection. Furthermore, we found that Tc1 cell responses were stronger in *S. japonicum* infected *Batf3* ^−/−^ mice. Opposed to previous studies, our findings suggest that during the infection of *S. japonicum*, the deletion of *Batf3* activates CD8^+^T cells to secrete IFN-γ and attenuate liver pathological damage in mice through immune regulation.

*Batf3* ^−/−^ mice can compensatorily produce a certain amount of CD8α^+^ DCs through *Irf8* transcription factor pathway, the newly generated CD8α^+^ DCs express Clec9A on the surface as wild type CD8α^+^ DCs, and have the same cross-presenting function [36]. Thus, CD8α^+^ DCs can be divided into *Batf3*-dependent (*Id2-Nfil3-Batf3*) and *Irf8-*dependent (*PU.1-Irf8*) groups. Ashok et al. [37] reported that *Batf3* ^−/−^ mice infected with *Leishmania major* lack CD8α^+^ DCs cross-presentation, lymphoid cells secreted less IFN-γ and more Th2 and Th17 cytokines, and had severe pathological damage and high worm burden. Sanchez-Paulete et al. [38] reported that because of the lack of cross-presentation activation of tumor antigens and new antigens associated CTLs to participate in immune response, immune regulation monoclonal antibodies lost their enhanced immunotherapeutic effects on tumor therapy in *Batf3* ^−/−^ mice. These studies suggest that cross-presentation of *Batf3*-dependent CD8α^+^ DCs in these models is critical. In 2015, Mott et al. [39] found that a small amount of CD8α ^+^ DCs could be detected in *Batf3* ^−/−^ mice infected with HSV-1, and HSV-1 latency situation in *Batf3* ^−/−^ mice was not different from wild-type mice, while latent infection was attenuated in BXH 2 mice (*Irf8* mutant). This study suggested that cross-presentation of *Irf8*-dependent CD8α^+^ DCs may play a critical role in HSV-1 infection. Cross-presentation of *Irf8*-dependent CD8α^+^DCs may have a key role in schistosomiasis infection.

*Irf8* is one member of the interferon regulatory factors family. *Irf8* binds to *PU.1* protein and forms a complex to regulate the maturation and function of DCs [40, 41]. In this study we detected a small amount of CD8α^+^ DCs in *Batf3* ^−/−^ mice infected with *S. japonicum*. Compared with wild-type B6 mice, *Irf8* and *PU.1* were highly expressed in the spleen cells of *Batf3* ^−/−^ mice. Collectively, our study demonstrated that in the absence of *Batf3*, a small amount of *Batf3*-independent CD8α^+^ DCs might be compensatorily induced in an *Irf8*-dependent manner. These newly generated CD8α^+^ DCs may have a more powerful function in cross-presenting and activate CD8^+^T to secret IFN-γ^+^ which can attenuate hepatic pathological damage in *Batf3* ^−/−^ mice.

## Conclusions

In summary, by using *Batf3* ^−/−^ mouse model of schistosomiasis japonica, we demonstrate the association of *Batf3* with the immunoregulation of the liver granuloma formation without affecting *S. japonicum* adult worm load and egg production, suggesting an important role for *Batf3* in regulation of Tc1 responses in schistosomiasis. We also demonstrated that without *Batf3,* mice will compensatorily generate *Batf3*-independent CD8α^+^DC. The function of this newly generated CD8α^+^DC needs to be further investigated. In addition, these novel findings imply that *Batf3* may function as a new therapeutic target if it is directly involved in modulating Tc1 cell responses for schistosomiasis and or other immune-associated diseases.

## Abbreviations

AOD: IOD/Area
APC: Antigen-presenting cell
*Batf3*: Basic leucine zipper transcriptional factor ATF-like 3
CD4: Cluster of differentiation 4
CD8: Cluster of differentiation 8
CD8^+^T(CTL): Cytotoxic T lymphocyte
FCM: Flow cytometry
IFN-γ: Interferon gamma
IL-4: Interleukin 4
IOD: Integrated optical density
SEA: Soluble egg antigen
Th1: Type 1 T helper lymphocytes
Th2: Type 2 T helper lymphocytes
